# Common hepatic duct necrosis following transarterial chemoembolization for hepatocellular carcinoma: A case report and literature review

**DOI:** 10.1016/j.ijscr.2025.111283

**Published:** 2025-04-12

**Authors:** Duc Tien Dao, Van Quynh Nguyen, Manh Thang Tran, Van Manh Nguyen, Duc Trung Le

**Affiliations:** a Oncology and Nuclear Medicine Center, Military Hospital 175, Ho Chi Minh City 70000, Viet Nam; b Department of Abdominal Surgery, Military Hospital 175, Ho Chi Minh City, 70000, Viet Nam; c College of Health Sciences, VinUniversity, Hanoi 113000, Viet Nam

**Keywords:** Transarterial chemoembolization, Hepatocellular carcinoma, Bile duct necrosis, Jaundice, Hepatectomy, Case report

## Abstract

**Introduction:**

Transarterial chemoembolization (TACE) is an effective hepatocellular carcinoma (HCC) treatment. However, it can lead to complications such as bile duct necrosis, which may cause severe morbidity and require complex management.

**Case presentation:**

A 58-year-old male with hepatitis B-associated HCC developed progressive jaundice and cachexia following TACE. Imaging revealed bile duct necrosis at the biliary bifurcation with intrahepatic duct dilation. Endoscopic and percutaneous interventions were considered but deemed inadequate due to the extent of bile duct injury, prompting surgical management with segment 4b segmentectomy and hepaticojejunostomy. This approach restored biliary continuity and alleviated symptoms effectively.

**Clinical discussion:**

Bile duct necrosis following TACE lacks standardized treatment protocols, posing therapeutic challenges. Surgery, though invasive, offers a definitive solution for extensive injury, especially when balanced against oncologic needs and patient comorbidities like cirrhosis. This case highlights the value of individualized, multidisciplinary strategies.

**Conclusion:**

Surgical intervention can be a viable option for bile duct necrosis following TACE when conservative measures fail. Early recognition and tailored management at specialized centers are critical for optimizing outcomes in this rare but severe complication.

## Highlights

• Bile duct necrosis following TACE is a rare complication.
• A high degree of clinical suspicion is needed to promt diagnosis and treatment.
• Surgery offers a definitive solution for extensive bile duct necrosis.

## 1 Introduction

Transarterial chemoembolization (TACE) is a well-established locoregional therapy for unresectable hepatocellular carcinoma (HCC), often used as a bridge to transplantation or for palliation [1]. While generally considered safe, TACE carries risks of complications, including post-embolization syndrome, hepatic infarction, spinal cord ischemia, and, less commonly, bile duct injuries [[2], [3], [4], [5]]. Bile duct necrosis (BDN) is a rare but potentially life-threatening consequence of TACE and arises from ischemic damage to the peribiliary arterial plexus. The disruption of bile duct integrity can lead to bile leakage, obstruction, infection, and, in severe cases, liver failure [[6], [7], [8]].

Although reports of BDN after TACE exist, optimal management strategies remain undefined. Conservative approaches, including endoscopic or percutaneous drainage, are often insufficient in severe cases, necessitating surgical intervention [7,9]. We report a rare case of BDN post-TACE managed surgically with hepatectomy and biliary reconstruction, achieving a favorable outcome. This report highlights the challenges of early diagnosis, the rationale for surgical intervention, and the importance of a multidisciplinary approach in managing this rare complication. This case was reported in accordance with the SCARE criteria [10].

## 2 Presentation of case

A 58-year-old male with a history of chronic hepatitis B underwent routine screening at another hospital, where abdominal CT scans revealed a 23 × 22 mm hypoattenuating lesion in segment 4 consistent with HCC. No vascular thrombosis was noted, and alpha-fetoprotein (AFP) was 2.89 ng/m. After consulting, he opted for undergoing TACE rather than hepatectomy, preferring a less invasive approach to manage his condition. The patient underwent TACE involving embolization of the tumor-feeding artery using 2 mL of Embozene Tadem 40 μm mixed with 50 mg of doxorubicin and gelatin sponge microparticles. Post-procedure, he reported mild right subcostal pain without fever or jaundice. A two-day follow-up CT scan showed an ill-defined hyperdense lesion with thickened gallbladder walls (9 mm) but no biliary anomalies. Discharged on day 6, he had persistent mild pain but normal laboratory findings.

On post-TACE day 10, severe right subcostal pain prompted readmission to a local hospital, where acute cholecystitis was diagnosed. Emergency laparoscopic cholecystectomy revealed a necrotic, inflamed gallbladder (4 × 8 cm) with thickened walls and subhepatic bile collection. Extrahepatic bile duct dilation and focal necrosis were noted but not addressed. He recovered and was discharged after 1 week.

**Fig. 1:**
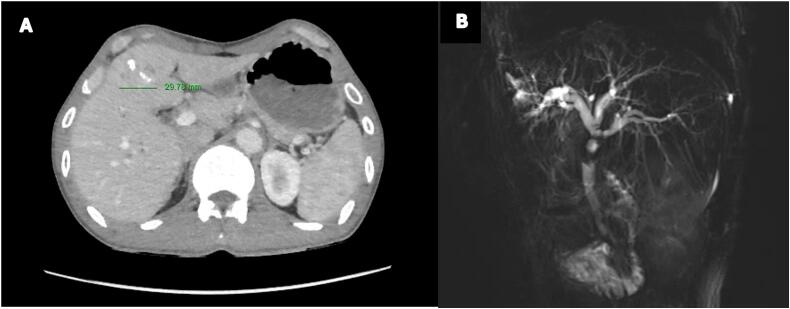
Pre-operative abdominal images: (A) Abdominal CT scan showed a tumor in the segment 4b with HCC characteristics; (B) Abdominal MRI showed the disruption at the biliary bifurcation combined with intrahepatic bile duct dilation.

**Fig. 2:**
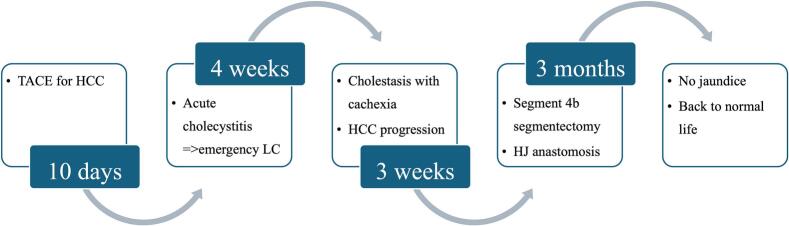
Clinical presentation timeline (TACE: Transarterial chemoembolization; HCC: Hepatocellular carcinoma; LC: Laparoscopic cholecystectomy; HJ: Hepaticojejuno).

Three weeks later, he presented to our hepatobiliary surgery unit with progressive jaundice, anorexia, cachexia (weight 43 kg, height 167 cm, BMI 17.56), and worsening epigastric pain. Laboratory tests showed cholestasis (direct/total bilirubin: 49.91/78.7 μmol/L) and elevated liver enzymes (AST/ALT: 88.9/116 U/L) without signs of infection or liver failure. Imaging confirmed HCC progression (22 × 28 mm) in segment 4b (Fig. 1A) and biliary bifurcation necrosis with intrahepatic duct dilation (Fig. 1B). Multidisciplinary consultation favored surgery over endoscopic or percutaneous options due to extensive necrosis and oncologic needs. Nutritional support preceded surgery to address cachexia. The timeline for this patient's clinical presentation is summarized in Fig. 2.

**Fig. 3:**
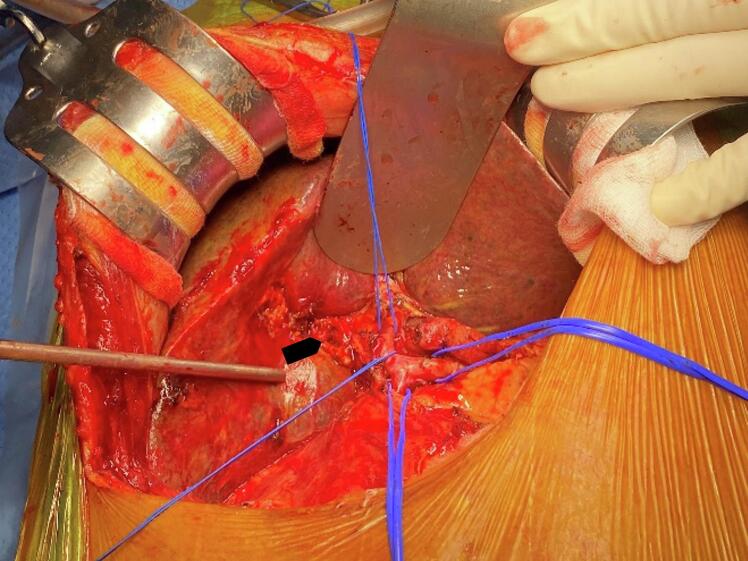
Intraoperative finding: The necrosis of the common hepatic duct (arrow).

**Fig. 4:**
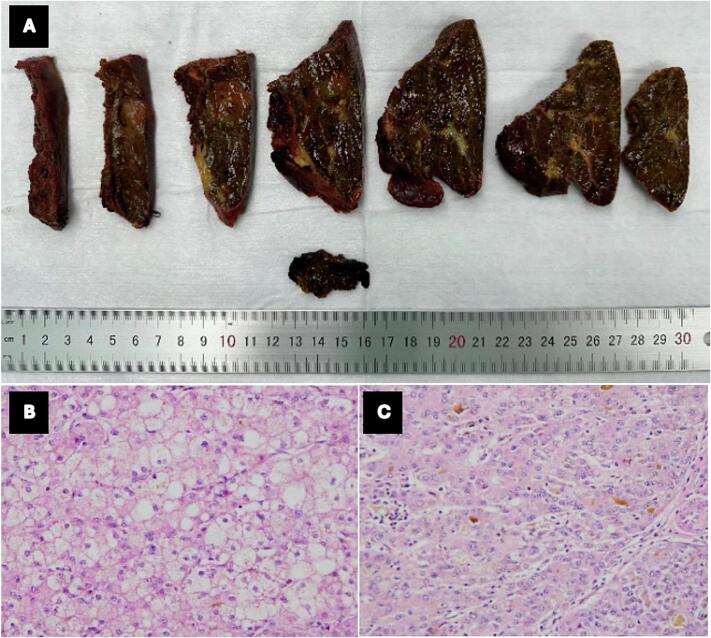
Pathological findings: (A) Macroscopic examination showed the tumor confined within the resection specimen with a clear margin and cholestasis of the liver parenchyma (B—C) Microscopic examination confirmed moderately differentiated HCC with underlying cirrhosis (H&E).

After three weeks, surgery via a right J-shaped incision revealed a 2 × 3 cm segment 4b tumor, an enlarged cholestatic liver, and a 2.5 cm necrotic common hepatic duct segment causing obstruction (Fig. 3). Segment 4b segmentectomy and hepaticojejunostomy were performed. On postoperative day (POD) 1, the patient was stable with mild abdominal distension, tolerating oral intake and mobilizing with assistance. By POD 7, a CT scan showed bilateral pleural effusions (right > left) and a 5 cm fluid collection at the liver resection site. Percutaneous drainage of 500 mL clear yellow pleural fluid resolved mild dyspnea and pleuritic pain. By POD 12, imaging and labs (direct/total bilirubin: 13.8/29.3 μmol/L, PT: 68 %, AST/ALT: 29/18.8 U/L) confirmed recovery, enabling drain removal and discharge on POD 13. Pathology confirmed moderately differentiated HCC with cirrhosis (Fig. 4).

At the 3-month follow-up, the patient was jaundice-free, had gained 10 kg (weight 53 kg), resumed normal activities, and had stable liver function.

## 3 Discussion

TACE is a mainstay for unresectable HCC, balancing efficacy with a low complication rate [[1], [2], [3]]. Biliary complications, including necrosis, occur in 0.9 %–12.5 % of cases, driven by embolic occlusion of the peribiliary arterial plexus, leading to localized ischemia [4,[11], [12], [13]]. This ischemia damages the biliary epithelium, enabling bile acid infiltration and necrosis, progressing to fibrosis, strictures, or complications like bilomas and cholestasis [6,8,12]. Interestingly, bile duct injury tends to increase as the size of blocked arteries decreases because the blood flow cannot be compensated by the development of collaterals or retrograde portal blood [13]. In this case, selective embolization using 40 μm particles likely exacerbated peribiliary ischemia as smaller embolics penetrate deeper into the microvasculature. The literature suggests that optimal embolic particle sizes range from 100 to 500 μm, with 100–350 μm preferred for conventional TACE and 100–300 μm for drug-eluting bead (DEB)-TACE, to achieve effective tumor embolization while minimizing non-target ischemia and complications like BDN [14,15]. The use of 40 μm particles, well below this range, likely heightened the risk in our patient. Necrosis predominantly affects the mid-common bile duct and the biliary bifurcation, as observed in this case when the necrosis extends 2.5 cm from the biliary bifurcation [13].

Predicting BDN following TACE remains challenging due to the absence of validated biomarkers. However, specific risk factors include tumors' proximity to major bile ducts, small embolic particle size, and underlying cirrhosis, all of which were present in our patient [16,17]. A recent study further identifies additional risk factors for bile duct injury post-TACE, including higher lipiodol dosage, use of gelatin sponge particles, selective embolization near biliary structures, and tumor hypovascularity, based on multivariate analysis of 847 HCC patients [17]. In our case, the use of gelatin sponge particles aligns with these findings. Moreover, postoperative increases in GGT and ALP (≥2 times preoperative levels) have been proposed as predictive indicators of bile duct injuries, offering a potential tool for early detection [17]. Symptoms evolved insidiously from mild pain to jaundice over 3 weeks, consistent with prior reports [8,13]. Early post-TACE imaging missed biliary changes, highlighting CT's diagnostic limitations. Initial misdiagnosis as cholecystitis delayed recognition, emphasizing the condition's rarity.

Managing BDN remains challenging, with no standardized treatment. Kobayashi suggests intervention for clinical decline, favoring endoscopic or percutaneous drainage initially [9]. These approaches are effective for accessible lesions, bilomas, or infection requiring decompression. However, in this case, extensive BDN at the biliary bifurcation and delayed presentation precluded endoscopic stenting, as the damaged segment was inaccessible for effective placement. For intrahepatic necrosis or patients with poor prognosis, a non-invasive approach with antibiotics and biliary drainage may required, particularly when bilomas or sepsis are present [6,12]. In our patient, the absence of biloma or infection eliminated the need for urgent drainage. Unlike cases requiring prompt decompression for sepsis, this patient showed no signs of infection, affording time to optimize his nutritional status and overall condition prior to definitive surgery. Hepaticojejunostomy and segmentectomy addressed both biliary obstruction and HCC, contrasting with conservative approaches suited for intrahepatic necrosis or poor prognosis patients.

In our literature review, previous reports of BDN following TACE predominantly manifested as bilomas often managed adequately with drainage and antibiotics [6,7,12,16]. In contrast, our case required biliary reconstruction due to extensive necrosis and obstruction, marking a novel approach. To our knowledge, this is the first report of BDN following TACE necessitating such surgical intervention. This case underscores the evolving role of surgery in managing TACE-induced biliary necrosis. While endoscopic interventions remain the cornerstone of early management, timely surgical resection and biliary reconstruction offer definitive solutions for select patients. Future research should focus on refining risk stratification models and optimizing embolization protocols to minimize biliary complications post-TACE.

## 4 Conclusion

BDN following TACE, though rare, demands prompt diagnosis and tailored intervention. This case demonstrates that segmentectomy and hepaticojejunostomy can effectively manage extensive necrosis and HCC when conservative options fail, offering oncologic control and biliary restoration. Multidisciplinary collaboration, nutritional optimization, and specialized care were pivotal to success.

## Credit authorship contribution statement

Concept, consent, literature review, drafting of the initial and final manuscript, approval of the final manuscript - All authors.

## Informed consent

That statement indicates that the patient whose case is being reported in the journal provided written consent before the case report and associated pictures were published. The purpose of obtaining informed consent is to ensure that patients are aware of the publication and any identifiable information that may be included. By obtaining written consent, the journal demonstrates its commitment to protecting the privacy and rights of the patient.

Additionally, the statement suggests that the journal's Editor-in-Chief has the authority to verify the existence of the written consent. If requested, they can examine a copy of the written consent to confirm that the patient obtained the necessary permission.

## Ethical approval

Our institution does not require ethical approval to report individual cases or case series.

## Guarantor

Van Quynh Nguyen M.D.

## Funding

The study did not receive external funding.

## Declaration of competing interest

We have no conflicts of interest to disclose.
